# Designing clinical practice feedback reports: three steps illustrated in Veterans Health Affairs long-term care facilities and programs

**DOI:** 10.1186/s13012-019-0950-y

**Published:** 2020-01-21

**Authors:** Zach Landis-Lewis, Jennifer Kononowech, Winifred J. Scott, Robert V. Hogikyan, Joan G. Carpenter, V. S. Periyakoil, Susan C. Miller, Cari Levy, Mary Ersek, Anne Sales

**Affiliations:** 1 Department of Learning Health Sciences, University of Michigan Medical School, 1161 J NIB, 300 N. Ingalls Street, SPC 5403, Ann Arbor, Michigan 48109-5403 USA; 2 VA Ann Arbor Healthcare System, Ann Arbor, Michigan USA; 3 VA Palo Alto Health Care System, Palo Alto, California USA; 4 Department of Internal Medicine, University of MichiganMedical School, Ann Arbor, Michigan USA; 5 Corporal Michael J. Crescenz VAMC, Philadelphia, Pennsylvania USA; 6 School of Medicine, Stanford University, Palo Alto, California USA; 7 Brown University School of Public Health, Providence, Rhode Island USA; 8 Eastern Colorado Health Care System, Aurora, Colorado USA; 9 School of Medicine, University of Colorado Anschutz Campus, Aurora, Colorado USA; 10 School of Nursing, University of Pennsylvania, Philadelphia, Pennsylvania USA; 11 Perelman School of Medicine, University of Pennsylvania, Philadelphia, Pennsylvania USA

**Keywords:** User-centered design, Audit and feedback, Clinical quality improvement, Long-term care, Goals of care

## Abstract

**Background:**

User-centered design (UCD) methods are well-established techniques for creating useful artifacts, but few studies illustrate their application to clinical feedback reports. When used as an implementation strategy, the content of feedback reports depends on a foundational audit process involving performance measures and data, but these important relationships have not been adequately described. Better guidance on UCD methods for designing feedback reports is needed. Our objective is to describe the feedback report design method for refining the content of prototype reports.

**Methods:**

We propose a three-step feedback report design method (refinement of measures, data, and display). The three steps follow dependencies such that refinement of measures can require changes to data, which in turn may require changes to the display. We believe this method can be used effectively with a broad range of UCD techniques.

**Results:**

We illustrate the three-step method as used in implementation of goals of care conversations in long-term care settings in the U.S. Veterans Health Administration. Using iterative usability testing, feedback report content evolved over cycles of the three steps. Following the steps in the proposed method through 12 iterations with 13 participants, we improved the usability of the feedback reports.

**Conclusions:**

UCD methods can improve feedback report content through an iterative process. When designing feedback reports, refining *measures*, *data*, and *display* may enable report designers to improve the user centeredness of feedback reports.

Contributions to the literature

- User-centered design methods offer an approach to improvement of audit and feedback, a widely used implementation strategy, but guidance on how to use these methods is lacking.
- We describe a novel method for the refinement of feedback reports to recognize dependencies in the audit process, involving practice measures (i.e., key performance indicators) and data, and their implications for information displays.
- In a national-scale initiative to design clinical practice feedback reports in long-term care settings in the Veterans Health Administration, the method yielded important learning and design changes that improved feedback report usability.

## Background

Feedback interventions are widely used [1], but evidence suggests we have limited knowledge of how and when these interventions positively influence clinical practice [2]. Theory and expert consensus support the idea that report design may affect the influence of feedback interventions on clinical practice [3–6]. Best practice guidance about designing feedback reports and dashboards recommends testing them with users (i.e., the people who receive reports and use them to change practice) [1, 3, 7–11]. When used in audit and feedback as an implementation strategy [12], the content of feedback reports depends on a foundational audit process involving performance measures and data, but these important relationships have not been adequately described.

User-centered design (UCD) refers to various methods for developing and testing human-created products (i.e., artifacts). These methods can enable feedback report designers to recognize defects and improvement opportunities in both the form of the report (i.e., how information is displayed) and its content (i.e., what information is communicated). For example, UCD activities improved the design of feedback reports for home healthcare professionals regarding report colors (form) and regional performance comparisons (content) [13]. By repeatedly testing an artifact, a designer can further refine the design of a prototype until no significant problems are identified [14].

UCD methods have been applied to feedback reports in home healthcare [13] and primary care settings [8, 15]. Colquhoun et al. [13] incorporated paper prototyping, interviews, focus groups, cognitive interviewing, and think-aloud methods across two design phases to optimize an audit and feedback intervention for home healthcare providers. Brown et al. incorporated interviews, video-based content analysis, eye-tracking analysis, and questionnaires to improve the usability of an electronic audit and feedback system [8, 15]. These studies demonstrate the potential contribution of UCD methods to feedback report design and highlight a range of methods that might improve the influence of feedback on clinical practice.

Our objective is to propose a method for user-centered design of feedback reports that recognize relationships between refinements to audit (i.e., measurement) processes and feedback display. To illustrate the method, we describe a feedback report design process for a national initiative to implement goals of care conversations (GoCCs) for veterans in long-term care facilities and programs within the Veterans Health Administration [16, 17]. The purpose of using feedback reports is to influence healthcare professionals and teams to adopt new practices and to identify opportunities for performance improvement.

## Methods

**Fig. 1:**
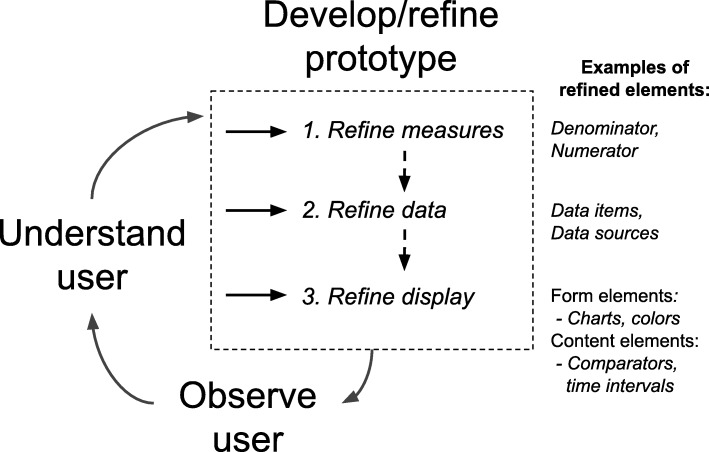
Three refinement steps in a user-centered design process [14] for feedback reports: refine measures, data, and display

We describe three key steps in a UCD process for feedback reports. The UCD process begins with understanding the user, and then proceeds through the three refinement steps for a prototype report’s *measures*, *data*, and *display*, followed by observation of the use of the refined prototype (Fig. 1). *Measures*, also called metrics, key performance indicators, or quality measures, are standardized processes for generally numeric assessment of the structure, processes, or outcomes of care [18, 19]. For each measure, *data* items are specified that originate from various sources, such as manual chart abstraction, administrative and billing systems, or an electronic health record system. *Measures* and *data* are organized in a feedback report containing design elements [4], including content and form elements, which we refer to as the report’s *display*.

### Understanding the user

Designing a useful and appropriate feedback report requires the designer to understand the purpose of the report, the people who will use it, and their context. Users are the intended recipients of the reports, including both frontline healthcare professionals and quality improvement managers. The role of a user is different from that of other project stakeholders, such as policymakers or administrators who may direct a program and decide to initiate a feedback intervention, but who do not receive feedback about their clinical practice.

Understanding how users are influenced by a report, especially regarding emotional responses, is essential for refining a prototype [20]. Suggested design methods for understanding the user are described in Additional file 1.

### Develop/refine prototype

Prototype feedback reports are sketches, drafts, or models of reports that typically contain artificial data and which provide enough information for a user to understand the proposed form and content of the report. Prototypes can be co-created with users or developed without user involvement prior to testing. Refinement of prototypes can be based on user feedback and suggestions during co-creation or based on requirements that explicitly outline constraints for the report. For example, requirements can be expressed as statements such as “reports must be printable in black and white” or can be expressed in the form of “user stories” that link a specific user role with a report characteristic and the purpose of the specific requirement [21, 22].

1. Refine measures: Refining measures is a process of changing performance calculations. Performance calculations are sums, averages, or rates, in addition to other less common calculations such as distributions, standard deviations, or more complex functions [23]. Changes to these calculations can include adding or removing a measure. One common change is to the inclusion or exclusion criteria for measure’s denominator (i.e., risk adjustment) to more accurately identify the patient population whose care can be improved. Refinement of a measure may require collection of additional reliability and validity evidence.
2. Refine data: Refinement of data is a change to the data items and sources used in the calculation of performance. Data refinement may be required by a refined understanding of the user directly or from refinement of a measure (Fig. 1). Changes to measures may result in a need to add or remove data items or sources. Data quality requirements may also lead to the refinement of data sources. System and team-level changes, such as new personnel, EMR software updates, and clinical workflow redesign, can affect data quality, requiring the refinement of data. Variation in practice across clinics or facilities may also require refinement of data.
3. Refine display: Refinement of a display is a change to the report’s content or form elements. Examples of display content include comparators (e.g., benchmarks, goals), time intervals shown in a performance history, and framing that relates performance to anticipated gains or losses. Form elements include charts, tables, and text. The appropriate display for a report can be impacted by refined measures and data, or from refined understanding of the user directly (Fig. 1). For example, the addition of a measure requires changes to the reports form and content to display new information. Refinements from understanding the user may relate to user preferences or ability to read a type of chart [24] or user expectations for meaningful comparators [25].

In early iterations of the prototype, “low-fidelity” [26] sketches can be created with contrasting features to yield insights into the usefulness of feedback report elements, including visual displays and text. To create sketches and generate ideas for reports, a collaborative design approach can be used by teams to produce and critique many ideas within a half-day design session [27]. A recommended activity for design of prototypes is to identify and specify the cognitive tasks involved in using the report [28, 29]. For feedback report prototypes, cognitive tasks include comparison of values between current performance and a comparator, and perception of a trend that shows a change in practice over time. Sub-optimal visualization of the information can mislead users or increase cognitive burden [30, 31]. For example, requiring users to do arithmetic or to process a large set of practice measures increases cognitive burden, which can result in users ignoring the report.

### Observe user

Observing users as they attempt to understand and interpret a report can reveal its defects. Usability testing is a form of user observation that involves preparing tasks and classifying the types of errors that occur as a result of using a prototype [32]. During observation, a facilitator may use an interview guide with questions about users’ comprehension and interpretation of visualizations or to assess appropriateness and acceptability. Suggested usability testing methods for observing the user are included in Additional file 1.

### Exiting the UCD cycle

Exiting the UCD cycle can happen when users have demonstrated that the feedback report is usable and that all of the identified user requirements are satisfied, including those relating to users’ perceptions and emotional responses to the report. Reaching this point enables the design team to confidently proceed to develop reporting tools or to deliver a design specification for report development.

## Results

We illustrate the proposed method through its application in a UCD study to design feedback reports as part of a broader, large-scale audit and feedback intervention study [16]. The intervention was funded by the VA Quality Enhancement Research Initiative program in support of the Life-Sustaining Treatment Decisions Initiative, a United States Veterans Health Administration initiative led by the National Center for Ethics in Health Care to promote high-quality goals of care conversations (GoCCs) and documentation of patients’ preferences for life-sustaining treatment [17, 33].

One key element of the Life-Sustaining Treatment Decisions Initiative is the use of a nationally standardized progress note and order set for documenting veterans’ goals of care and life-sustaining treatment decisions in the VA’s electronic health record system. The progress note and order set may be written in any Veterans Health Administration care setting (e.g., outpatient, inpatient, nursing home) and are viewable and durable across settings. Both the notes and orders are accessible via the VA’s Corporate Data Warehouse. Additional data relevant to report generation, such as a veteran’s admission status and the location of the GoCC, are also available in the data warehouse, creating a set of historical data elements that are available for practice measurement.

### Setting

This work was done in five long-term care Veterans Health Administration sites in the Western and Midwestern regions of the USA. Four of the sites were participating in the Life-Sustaining Treatment Decisions Initiative as demonstration sites that had implemented the initiatives’ progress note and order set. One site was added for the convenience of in-person meetings with the project team, where a key individual involved as a user was also a member of the research team. Long-term care settings and services at sites included both community living centers, which are VA-owned nursing homes, and home-based primary care programs to which eligible veterans were admitted for care provided in their homes by visiting healthcare professionals. All five sites had a home-based primary care program, while only four of the sites had a community living center facility (Table 1).

**Table 1:** Participating site facility and program characteristics

Characteristic	Community living centers (*N* = 4)	Home-based primary care programs (*N* = 5)
Median estimated Full-time equivalent (FTE) in 2016	--	--
Registered Nurses	26.6 (min 24.4, max 55.0)	4.3 (min 1.2, max 7.0)
Nurse Practitioners	--	0.9 (min 0.0, max 3.1)
Physicians - full time	0.5 (min 0.2, max 3.1)	0.1 (min 0.1, max 0.5)
Social Workers	--	1.5 (min 0.8, max 2.6)
Median estimated average daily patient census in 2016	45 (min 21, max 116)	99 (min 72, max 164)

### Participants

Participants were healthcare professional managers and administrators. One or more participants at each site was a designated “site champion” who had been identified by the VA National Center for Ethics in Health Care to lead and coordinate implementation efforts for the Life-Sustaining Treatment Decisions Initiative. Site champions from the 5 facilities recommended a total of 13 individuals, including themselves, to participate in the design process. The number of participants from each site ranged from 1 to 4. This interdisciplinary group of participants included 2 nurses, 1 nurse practitioner, 3 physicians, 2 quality improvement/compliance professionals, and 5 social workers.

### Application of the method: the UCD cycle applied to feedback report design

We conducted 12 iterations of the UCD cycle over approximately 18 months. We describe the method through its application over the 12 cycles that led to the design and development of feedback reports for community living centers and home-based primary care programs.

#### Understand user—initial cycle

**Fig. 2:**
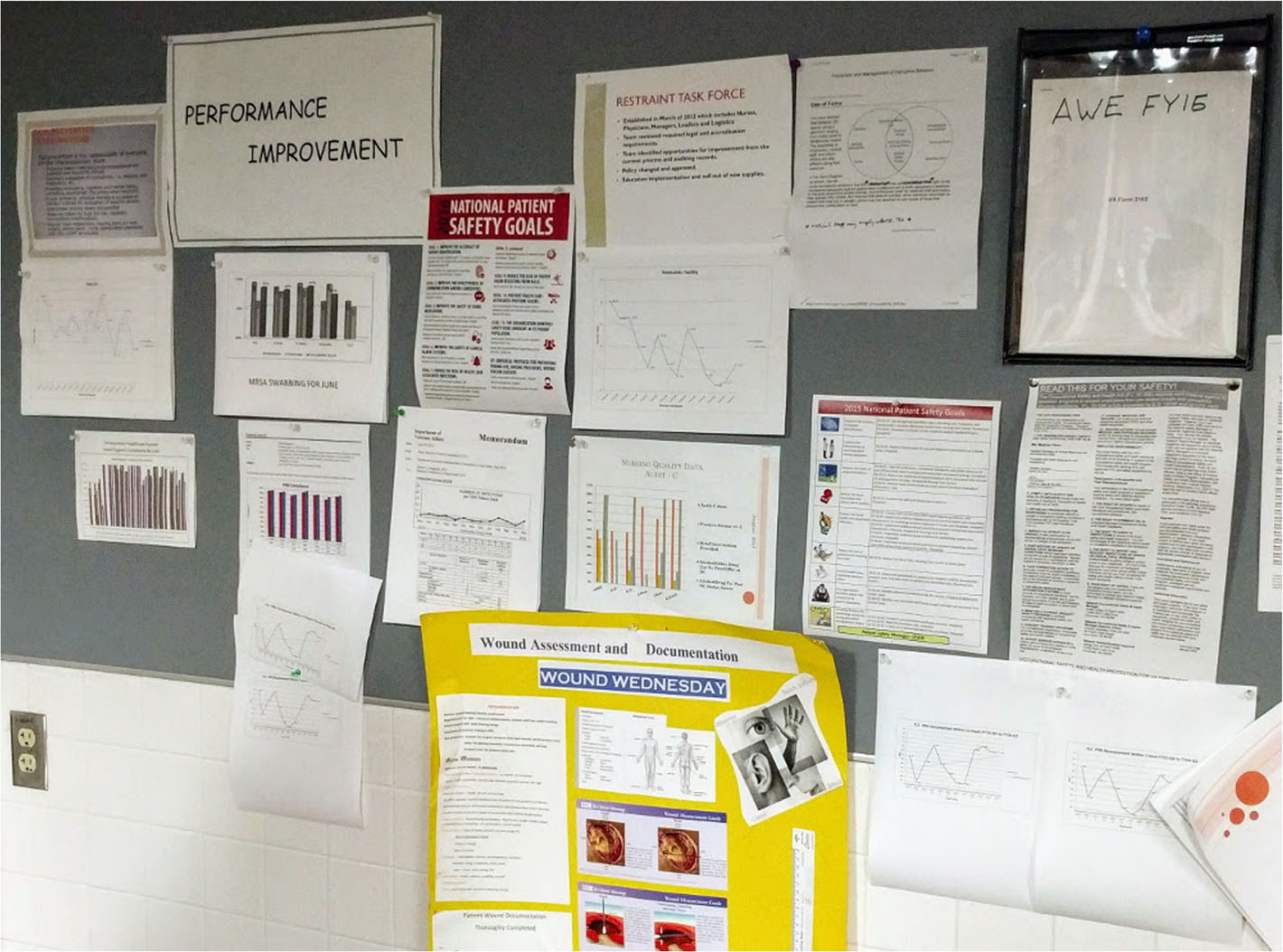
Example of a bulletin board that is used to post feedback reports

We visited all 5 sites to meet with participants. During the site visits, we interviewed participants, toured facilities, and met with other staff involved in implementing the Life-Sustaining Treatment Decisions Initiative to understand the context, professional roles, veterans’ care processes and environments, and the activities involved in conducting GoCCs and using the progress note and order template. We took field notes during interviews and reviewed them in group discussions about the context of participants’ work, including when GoCCs occur, where routine practice feedback is delivered, and to identify potential opportunities to measure documentation of GoCCs that could be summarized in feedback reports. With permission, we photographed bulletin boards on which practice feedback reports were routinely posted to capture characteristics of reports that healthcare professionals were accustomed to receiving (Fig. 2).

After each site visit, we discussed our observations in team meetings. We reviewed our notes and had monthly calls with the larger study team in which we discussed requirements and their implications for the practice measures, data, and visual displays in a report. We compared and contrasted characteristics of site contexts and participants to identify generalizable traits of users across sites and to identify contextual differences that reports would need to accommodate. The key differences that reports were adapted to accommodate were the intent to disseminate feedback reports and interest in facility-level comparison, which varied across sites (Additional file 2). These requirements also included channels for delivery of feedback (e.g., use of email, bulletin board posting). This initial step occurred over 7 months, during which we used meeting discussion notes to identify and develop preliminary measures, data, and displays.

#### Understand user—subsequent cycles

After completing the site visits, we conducted 12 iterations of the UCD cycle. Each iteration involved the usability testing of the prototype with multiple users (Table 2) and resulted in a refined understanding of users and changes to the prototype reports. We used anecdotes from our observations to support several types of refinements in our understanding, including recognizing false assumptions and contrasting user needs and preferences (Table 3 and Additional file 2, Example 1). The design team referenced these anecdotes, described in our discussion notes, when considering changes to measures, data, and displays.

**Table 2:** Example usability testing and interview guide for prototype report testing

Interview stage	Example questions/prompts
Introduction questions/rapport building	● Could you tell me about your role at the [participant’s facility name]? ● [Phone interviews] Are you in a place where you can view the first report? ● Do you have any questions before we look at the first prototype report?
Viewing 1st report prototype	● What are your initial reactions to this report? ○ [If no verbalizations are occurring] What are you thinking? ○ Listen and repeat back key points with rationales to check for understanding ● Comprehension tasks: ○ In what quarter was the percentage of Veterans with GoCCs highest? ○ What level of performance does the display show? ● Interpretation of the primary comparison in the report (e.g. a benchmark): ○ This chart contains a green line that represents average performance of other facilities in your region. Is that a meaningful comparison for you? If not, what would be meaningful? ● Time ranges: ○ What is the optimal reporting interval/timing for this report? Monthly? Quarterly? ○ What range of dates would you prefer to see? 1 year? more? less? ● Language and terminology: ○ The report uses the phrase "conversations documented" - does that make sense? If not, how would you say that? ● Organizational structure: ○ Are there organizational or team divisions that we could differentiate to better show this information?
Viewing subsequent report prototypes	● [Repeat questions from 1st report prototype] ● Report comparison: ○ Do you have a preference for seeing the data in one of these reports over another? ○ What are the characteristics that you prefer, and why?
Wrap-up	● Are there any other thoughts you would like to share, or any suggestions at all that you have for us today?

**Table 3:** Example refinement of prototype measures, data, and display about documenting goals of care conversations for VA providers in long-term care settings

Initial prototype report	Observe user	Understand user	1. Refine measure	2. Refine data	3. Refine display
*Key assumption:* Timeliness of goals of care documentation is important because admission to long term care reflects health status changes that may change a patient’s care goals. *Measure*: *Denominator:* Veterans newly admitted in quarter *Numerator:* Veterans newly admitted in quarter and having completed documentation within 7 days after admission *Data:* Admissions data for long term care facilities *Display:* Text and a bar chart showing percentage as a rate with sums for the numerator and denominator over recent quarters (Fig. 3).	Users expressed concern that the numerator’s 7-day window excluded Veterans with appropriate documentation from a prior admission. Users expressed that performance feedback about goals of care documentation at any time for newly admitted Veterans would be valuable.	Users value information about the historical reach of goals of care documentation.*Requirement:* At least one measure should reflect historical documentation of goals of care.	Include Veterans who have ever had goals of care documentation in the numerator.	Retrieve historical goals of care documentation data from the Corporate Data Warehouse from any prior date, and up to the time of generating the current report.	Revise the bar chart to reflect changes to the measure and data (Fig. 4, top).
		Users value timeliness information before and after the 7-day window. *Requirement:* At least one measure should show timeliness of documentation before and after admission.	Create a new measure to assess the timeliness of goals of care documentation for newly admitted Veterans. Create three new numerators: 1) Documented between 8 to 30 days following admission, 2) Documented within 7 days following admission, 3) Documented any time prior to admission.	Retrieve historical goals of care documentation data from the Corporate Data Warehouse in three separate time windows, reflecting the new numerators.	Develop a new chart that shows the count data for each numerator during each quarter (Fig. 4, bottom).
	Users did not expect that all Veterans admitted to their facilities were required to have a goals of care conversation because many are not seriously ill. Users expressed that separation of measures by short stay and long stay services was an acceptable proxy for serious illness status.	Users perceive priority of goals of care documentation to be high only for Veterans who are seriously ill. *Requirement:* Measures should reflect the Veteran populations’ severity of illness.	Divide all measure denominators into two parts, for Veterans who are seriously ill, and those who are not.	Use new data sources: Admitting service categories of short stay and long stay as a proxy for serious illness.	Develop a new chart to show documentation for short stay and long stay admissions (Fig. 5).

#### Develop/refine prototype

As a team, we processed a wide range of issues and considerations, including participants’ work, beliefs, priorities, and perceptions; barriers to anticipated change processes, data quality issues, technical limitations, and stakeholder priorities. Refinements varied, with some implicating minor changes to the display, and others implicating all three-prototype refinement steps (Additional file 2, Example 2). In some cases, there was a need to resolve conflicting requirements expressed from participants. For example, some sites requested regional comparison data while others requested to have no comparators in the report, requiring us to identify the most appropriate solution for all stakeholders, which was a report that did not use comparators, such as benchmarks or organizational performance goals.

##### Refine measures

Based on the requirements we identified in our initial site visits, we created ratio-based practice measures for GoCC documentation. The initial measures used counts of veterans newly admitted to community living centers or home-based primary care for a denominator. For a numerator, we used the count of those newly admitted and having GoCC documentation, yielding a percentage of newly admitted veterans who had a completed template within 7 days of admission. As a result of continued testing, we modified the measures. For example, participants expressed concerns that although measurement of GoCC timeliness was appropriate, it was at a lower priority than increasing the reach of documentation at any time in the patient population. In response to these concerns, we created a new measure addressing the historical reach for documentation of GoCC (Table 3). We further refined measures by broadening the time windows for timeliness-focused measures. This change enabled the reports to display when conversations had ever been documented prior to admission and up to a full 30 days after admission to provide more information about conversation timing in community living centers. Similar changes were introduced for home-based primary care program reports. In later cycles of report testing with refined measures, participants confirmed that the measures were appropriate and raised no further significant concerns (Additional file 2, Example 3).

##### Refine data

Refinement of measures required refinement of data items from the Corporate Data Warehouse. This process involved confirmation of data items and their appropriateness for use in new measures. For example, to support refined measure for the GoCC reach in community living centers, we needed to differentiate patients who were more likely to be seriously ill from those who were not (Table 3). We identified the admitting service (short stay or long stay) as a data item that adequately served as a proxy for severity of illness that participants could accept. Veterans admitted to long stay services in community living centers were recognized as likely seriously ill and at higher priority for having a GoCC than those admitted to short stay services, such as for physical rehabilitation (Additional file 2, Example 2).

##### Refine display

**Fig. 3:**
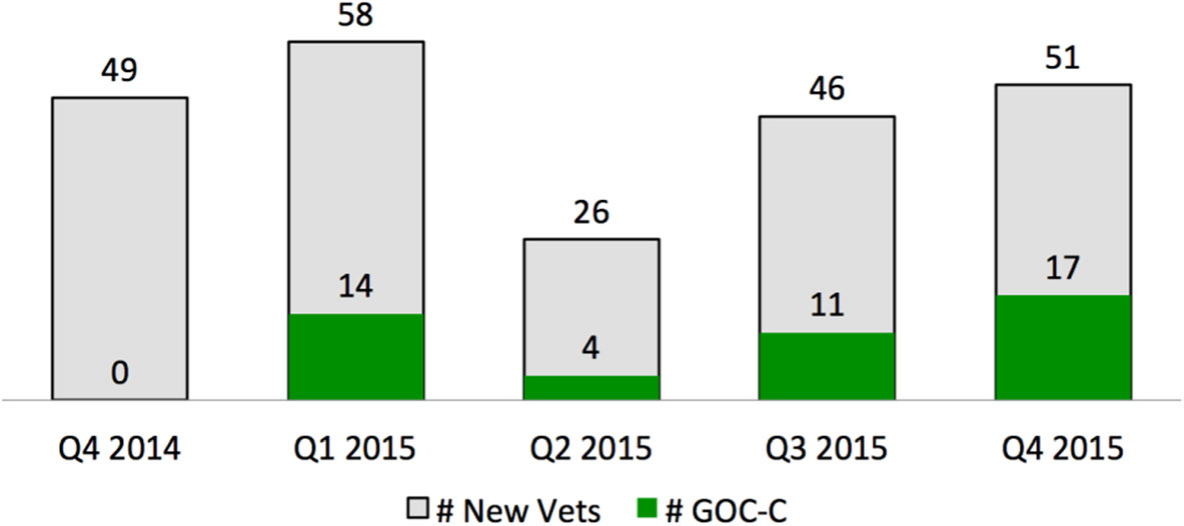
Prototype displays, version 1, that summed only conversations documented within 7 days. CLC, community living center

**Fig. 4:**
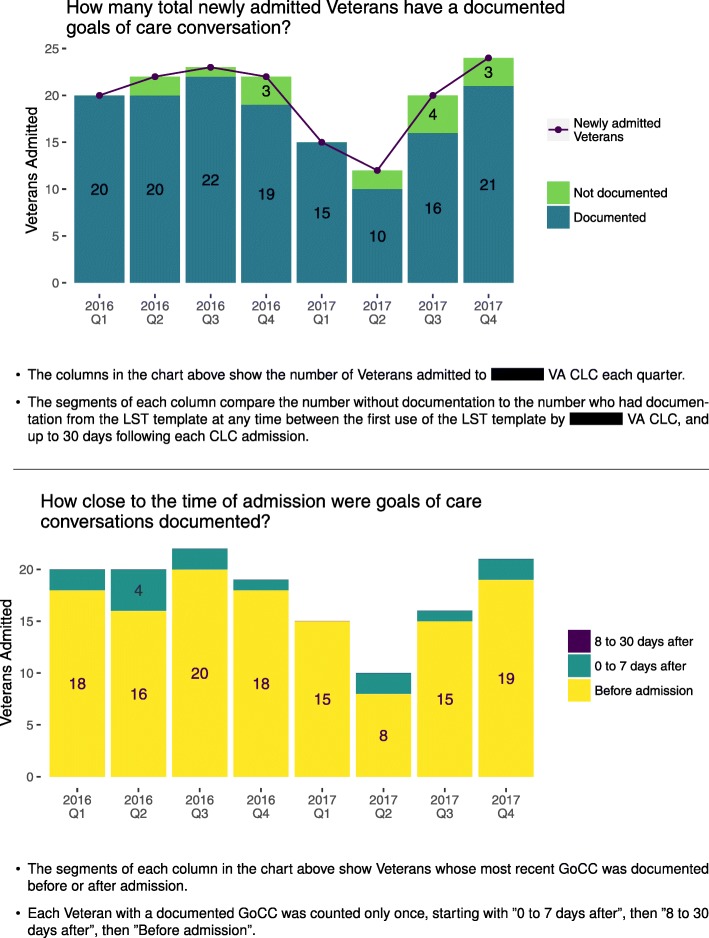
Prototype displays, version 12, that summed conversations ever documented (top) and the timeliness of documentation (bottom). CLC, community living center; LST, life-sustaining treatment

**Fig. 5:**
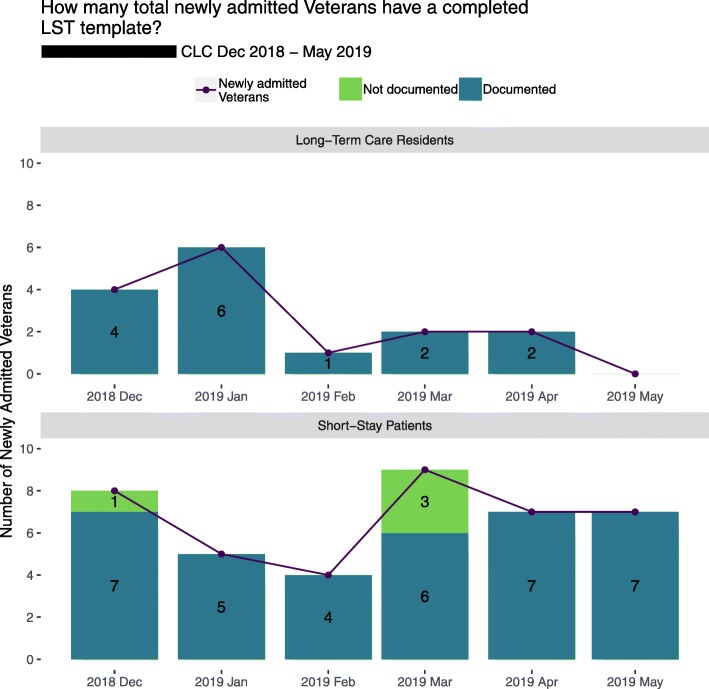
Chart from feedback report in June 2019 with refined measures addressing admitting service (short stay vs. long-term care). CLC, community living center; LST, life-sustaining treatment

We created prototype charts and drafted report text to display practice data. We used graphical displays of practice data as a focus of the report which could potentially be used in electronic or paper form and delivered via handout, email attachment, or bulletin board posting. In the initial cycle of the design process, we created charts in a spreadsheet and document editor as low-fidelity prototypes (Fig. 3). By the end of the 12th cycle, the report design featured two charts which alternately emphasize the reach and timeliness of documentation (Figs. 4 and 5).

#### Observe user

We conducted usability testing in 14 sessions (6 in-person meetings and 8 phone interviews) with 11 of the 13 participants. Interviews occurred over 7 months. During interviews, we asked participants to think aloud when viewing reports and gave them tasks in the form of questions that required them to interpret the data in the report (Table 3). We used a qualitative approach to usability testing [32], simply capturing key observations in notes. We opted to use phone interviews because participants worked at several locations and because our usability tasks were primarily based on the interpretation of non-interactive reports.

Usability testing generated insights in each of the three design steps. For example, some participants raised data quality issues, such as the accuracy of the denominator, which led to further refinement of data. Presentation format issues were raised, such as expressing a preference for seeing data presented both as counts and as percentages so that providers could better assess data accuracy. Participants also expressed a preference for bright colors to attract attention when reports were posted on a bulletin board. These and other observations were captured in notes for each call and reviewed in group discussions to interpret and refine our understanding of the participants in subsequent cycles.

#### Exiting the cycle

After 12 iterations, we transitioned from a prototyping phase to a software development phase and began producing reports for the 4 demonstration sites (Additional file 3).We also sought additional feedback that would allow for minor improvements to the report design. At the conclusion of the UCD phase, we began sending reports to new VA facilities that did not participate in the design process, as part of the larger project. Reports were generated each quarter and sent to site champions via email for further internal distribution. As of July 2019, quarterly reports had been delivered to more than 23 facilities or programs. In the 2 years following report, implementation we routinely requested site feedback about the usefulness of the reports, but have not conducted further usability testing. Multiple responses have led to ongoing minor changes and additions to the report, such as shifting the report timing from quarterly to monthly, and adding the provision of patient list data to complement the report data. We plan to provide ongoing maintenance support and have maintained software for the reporting tool in a public code repository (https://github.com/Display-Lab/goals-of-care).

## Discussion

We have described a proposed method for the UCD of feedback reports that follow refinement steps of the reports’ measures, data, and displays. We used the proposed method to identify feedback report requirements and to iteratively change the report design to increase its usability. Using this method, we identified divergent project stakeholder perspectives to arrive at a final design, including decisions about both its content and form that was acceptable to stakeholders and usable for participants.

Best practice guidance recommends the use of UCD to develop reports, including testing the recommended best practices themselves [3]. Studies of UCD applied to feedback report development are limited, but have demonstrated the use of iterative prototyping and user observation [8, 13]. We build on these studies to contribute a method that incorporates key feedback report components and their dependencies.

We propose a method that may serve as a general process for UCD of feedback reports, to implement and test best practice recommendations along with other insights gained from testing reports with users. The method specifies steps within a general UCD cycle [14] (understand user, develop/refine prototype, and observe user) and operationalizes the cycle to include 3 steps during prototype refinement: *measures*, *data*, and *display.* The transition from the *understand user* step to the *develop/refine prototype* step involves decisions for 3 alternate pathways (Fig. 1). The 3 steps have dependencies, such that *display* depends on *data*, which depends on *measures*. Changes to *measures* therefore are likely to necessitate subsequent refinement of *data* and *displays*.

We have illustrated this method with a feedback report design process in a large VA quality improvement project. We used the UCD cycle to iteratively improve the design of reports, based on the observed use of and reactions to feedback report prototypes by healthcare professionals. Improvements were made by identifying and reducing errors associated with the report design, both for interpretation of the data and in the appropriateness of the report for a given context, such as using peer-based comparators.

Across the 12 iterations through the UCD cycle, new requirements shifted from a tendency to address *measures* towards *displays*. In earlier report tests, participants frequently raised concerns about measures that were fundamental to the subsequent report design steps. Later in the testing of reports, measurement issues were less common, and refinement was focused on improving the text and charts in the report.

The proposed method enabled us to identify requirements that differed across participants and across the VA. In some cases, preferences for reports were contradictory, preventing us from designing a single optimal report for all facilities and programs. Key differences expressed were in the use of comparators in reports and in the expressed intention to disseminate the report among staff. Based on these observations, we anticipate that optimal feedback may need to be adaptable to recipient differences, allowing for a choice of alternate measures and corresponding displays.

The method we describe has implications for best practice in feedback interventions. We call attention to the maturation of performance measures as an important factor in the success of feedback interventions. For example, measure revisions resulted directly from report testing with long-term care providers, who indicated that the timeliness of GoCC and treatment preference documentation was a lower priority than reach (i.e., the spread of documentation practice in the veteran population). Given the unique contextual factors that appear to affect the appropriateness of measures, we anticipate that such adaptation of measures is a potentially important initial step in any feedback intervention.

An additional implication is that feedback reporting projects should consider allowing additional time to refine reports with users to ensure that reports will be useful and appropriate for influencing practice. Finally, an implication for the broader adoption of UCD in feedback report design would require better description of feedback report content and form, to enable improved evidence generation across research networks, such as the Audit and Feedback Metalab [34]. We recommend that work to better describe and define the components of feedback reports and their relationships be prioritized to enable better learning from feedback interventions that have been designed for specific user populations.

### Limitations

The proposed method emerged as we progressed through the UCD cycles that were applied to our project; therefore, they may not generalize to other design projects. For example, the method we describe may be most relevant to the development of novel measures, or their application to a new population, as more refinement and changes resulting from measure development will trigger more or different data needs and report development. In an intervention using standardized measures that cannot change over the duration of the intervention, such as with Healthcare Effectiveness Data and Information Set measures [35], the proposed measure refinement step may be less relevant. However, standardized measures may nevertheless be optimized for policymakers’ and organizational leaders’ information needs that differ from the information needs of healthcare professionals who receive feedback about their practice, especially at the facility or organizational level. As such, a key lesson is that standardized measures may require alignment with frontline providers’ information needs in order to be useful for improving the quality of care.

In applying UCD to the development of the feedback reports, our identification of divergent stakeholder perspectives resulted in design choices that reflected the trade-offs we encountered in terms of technical feasibility, available resources, stakeholder interests, best practice guidance, and evidence about audit and feedback. We anticipate that UCD methods hold potential to increase the effectiveness of reports, but recognize that this is an area in which evidence is lacking, and hence a potentially important area for future research.

We did not quantitatively assess the impact of using the proposed method. Further evaluation is needed to understand the impact of the proposed method on feedback report usability and uptake.

We did not assess the cost of using the method, in particular, the use of a design team with implementation science experts. The method’s value could perhaps be realized at low cost by a single individual, rather than involving a design team.

In our application of the proposed method, phone-based usability testing reduced the information we could perceive from participants’ facial expressions and body language that might inform emotional aspects of participants’ comments. Usability testing in an in situ or naturalistic work environment setting for healthcare professionals is likely to support better fidelity to the cognitive processes of perception and comprehension resulting from interaction with reports [28, 36]. Phone-based testing was perhaps more feasible because the prototypes we tested were single-page, non-interactive PDF documents that did not require navigation of interface controls or a sequence of actions to perform, which reduced participant’s task complexity.

We did not capture participant demographic data or assess participant diversity. We experienced challenges in arranging face-to-face interactions with extremely busy clinicians and coordinating schedules in this multi-site, large-scale project.

We believe that specializing the methods of UCD for feedback reports will make it more feasible for implementation researchers to use this method. We describe steps for operationalizing the “develop/refine prototype” step of the UCD cycle for feedback report design. Future research could additionally explore the operationalization of the UCD cycle for other steps of the cycle, which we have illustrated with examples.

## Conclusions

UCD methods can improve the usability of feedback reports through an iterative cycle of understanding users, developing prototypes, and observing interactions. When designing feedback report prototypes, using the key steps of refining *measures*, *data,* and *displays*, and planning to take time for this process may enable report designers to better translate users’ needs into report design changes. In a national-scale initiative to design clinical practice feedback reports for long-term care settings in the Veterans Health Administration, this method yielded important design changes and insights. These types of systematic approaches may improve the ability of feedback interventions to influence the provision of high-quality care for patients and their families everywhere.

## Supplementary information

**Additional file 1.** Example design techniques. Additional examples of design techniques that can be used for understanding and observing users of feedback reports.

**Additional file 2.** Examples from long term care. Additional examples of the application of the method in long term care facilities and the design team roles and responsibilities.

**Additional file 3.** Software development. Software development process used following the application of the proposed method.

## Data Availability

Because this work is being done as quality improvement, data will only be available from the authors on request and after approval by the authorizing officials. Publicly developed materials for this work are available in a GitHub repository: 10.5281/zenodo.1300783.

## Abbreviations

GoCC: Goals of Care Conversation
UCD: User-centered design
VA: Veterans Affairs
