# Supplementary material for: Designing clinical practice feedback reports: three steps illustrated in Veterans Health Affairs long-term care facilities and programs
Source: Implement Sci. 2020 Jan 21;15:7. doi: 10.1186/s13012-019-0950-y (PMC6975062; doi:10.1186/s13012-019-0950-y)
Supplement: Supplementary file 1 — Additional file 1. Example design techniques. Additional examples of design techniques that can be used for understanding and observing users of feedback reports. [file 13012_2019_950_MOESM1_ESM.docx]

**Additional file 1: Example design techniques**

*Understanding the user*

For a feedback report to influence practice, the user is likely to go through several cognitive steps: *viewing*, *comprehending*, *accepting*, and *forming an intention to act* on the feedback[1]. Techniques to understand users’ perspectives and needs include individual and focus group interviews[2,3] and observations of practice[4,5]. These qualitative approaches enable designers to acquire rich information about how feedback reports interact with users’ goals, preferences, problems of interest, and information needs.

Designers must also understand and address emotional responses to prototype reports[6]. Discouragement, happiness, anxiety, and relief are common responses to feedback[7]. Emotional expressions can be cues about users’ goals, preferences and contextual factors that may prevent a feedback report from being successfully delivered and acted upon. For example, in some contexts, peer comparison is desired and motivating, whereas in other contexts, such comparisons may result in discouragement or fear[8], such that clinical leadership may intervene to prevent subsequent reports from being delivered.

*Contextual inquiry*

One recommended approach for gaining an understanding of the user is contextual inquiry, a design method for data collection and focused learning about a work setting and user activities[5]. Contextual inquiry can be done by conducting 10-12 interviews combined with observations of report use for a setting, followed by interpretation of the data collected. Interviews and observations should cover the breadth of the users’ professional roles, such as providers (e.g., physicians, nurse practitioners), social workers, nurses and other team members. Ideally, user groups represent management and frontline clinical roles, especially in projects that deliver team and facility-level feedback where managers are the initial feedback report recipients. Contextual inquiry requires the design team members to take the role of an apprentice whose goal is to learn about practice as it happens, recognizing problems as opportunities to improve. The design team may aim to learn both about the practice to be implemented and organizational precedents for feedback reports.

Thematic analysis can be used to identify key issues with feedback report design. During interviews and observations, a member of a design team takes notes and may record audio if appropriate. Following interviews, the design team may transcribe audio and review notes, using reflection to identify and interpret key themes or issues. Ideally, an interpretation session is held by the team to create models and scenarios of the user and context that will be refined in subsequent cycles[5]. These models may be created as diagrams of the workflow, concept maps, or personas. Scenarios can be written to give examples of how reports are used and describe related assumptions held by a design team[9].

*Observing the user*

Usability testing can be done quantitatively or qualitatively, ranging from a highly structured process in which errors are documented by severity, to an unstructured process of note-taking to describe issues identified by the observer[10]. A technique that is especially relevant for studying cognition and can reveal errors in the report design is the “think aloud” method[10,11], in which users are asked to verbalize their thoughts as they comprehend and react to the report.

An option for usability testing is to use a comparative approach, inviting users to reflect on their preferences and experiences with multiple prototypes relative to one another as a means to elicit their rationale for preferences and to feel comfortable critiquing undesirable aspects of reports. Paper prototypes or digital sketches that suggest an unfinished design can also enable the user to more comfortably critique the reports[12]. Usability testing is commonly done in person but can also be done remotely[11,13]. Evaluating documents that are not interactive can be done via phone[11] when the primary method of data collection is a transcript of the user expressing their comprehension and interpretation of a feedback report using think aloud technique.

**References**

1. Ilgen DR, Fisher CD, Taylor MS. Consequences of individual feedback on behavior in organizations. J Appl Psychol. 1979;64(4):349–71.

2. Colquhoun HL, Sattler D, Chan C, Walji T, Palumbo R, Chalmers I, Jokhio I, Ivers NM. Applying User-Centered Design to Develop an Audit and Feedback Intervention for the Home Care Sector. Home Health Care Manag Pract. 2017 Aug 1;29(3):148–60.

3. Part Three: Three Strategies for Continuous Improvement of Physician Feedback Reporting Systems | Agency for Healthcare Research & Quality [Internet]. 2016 [cited 2018 Oct 4]. Available from: https://www.ahrq.gov/professionals/clinicians-providers/resources/confidreportguide/three-strategies.html

4. Cooper A, Reimann R, Cronin D, Noessel C. About Face: The Essentials of Interaction Design. John Wiley & Sons; 2014. 1112 p.

5. Holtzblatt K, Beyer H. Contextual Design, Second Edition: Design for Life. 2 edition. Cambridge, MA: Morgan Kaufmann; 2016. 530 p.

6. Roberts JP, Fisher TR, Trowbridge MJ, Bent C. A design thinking framework for healthcare management and innovation. Healthcare. 2016 Mar 1;4(1):11–4.

7. Kluger AN, Van Dijk D. Feedback, the various tasks of the doctor, and the feedforward alternative. Med Educ. 2010 Dec;44(12):1166–74.

8. Van Dijk D, Kluger AN. Feedback Sign Effect on Motivation: Is it Moderated by Regulatory Focus? Appl Psychol. 2004 Jan 1;53(1):113–35.

9. Rosson MB, Carroll JM. Usability Engineering: Scenario-Based Development of Human-Computer Interaction. 1st ed. Morgan Kaufmann; 2001.

10. Krug S. Rocket Surgery Made Easy: The Do-It-Yourself Guide to Finding and Fixing Usability Problems. 1 edition. Berkeley, CA: New Riders; 2009. 168 p.

11. Grudniewicz A, Bhattacharyya O, McKibbon KA, Straus SE. Redesigning printed educational materials for primary care physicians: design improvements increase usability. Implement Sci IS. 2015;10:156.

12. Buxton B. Sketching User Experiences: Getting the Design Right and the Right Design. 1 edition. San Francisco, CA: Morgan Kaufmann; 2007. 448 p.

13. Bastien JMC. Usability testing: a review of some methodological and technical aspects of the method. Int J Med Inf. 2010 Apr 1;79(4):e18–23.

14. Sales AE, Ersek M, Intrator OK, Levy C, Carpenter JG, Hogikyan R, Kales HC, Landis-Lewis Z, Olsan T, Miller SC, Montagnini M, Periyakoil VS, Reder S. Implementing goals of care conversations with veterans in VA long-term care setting: a mixed methods protocol. Implement Sci. 2016 Sep 29;11:132.
