# Supplementary material for: Designing clinical practice feedback reports: three steps illustrated in Veterans Health Affairs long-term care facilities and programs
Source: Implement Sci. 2020 Jan 21;15:7. doi: 10.1186/s13012-019-0950-y (PMC6975062; doi:10.1186/s13012-019-0950-y)
Supplement: Supplementary file 2 — Additional file 2. Examples from long term care. Additional examples of the application of the method in long term care facilities and the design team roles and responsibilities. [file 13012_2019_950_MOESM2_ESM.docx]

**Additional file 2: Examples from long term care**

*Example 1: Understanding users*

We asked site champions which professional roles they anticipated that feedback report users held, so that we could identify appropriate participants for report usability testing. Site champions responded differently, helping us to understand the range of users and revealing some false assumptions held by the design team. One site champion expressed an intention to disseminate the report widely, identifying multiple professional roles for us to consider, which broadened our understanding of the potential users with whom we needed to test the reports.

Another site champion who was a) highly engaged in the initiative and b) who had demonstrated high commitment to implementing GoCCs, expressed a clear position that feedback reports were not likely to be disseminated in any form at the site and that no comparisons to other facilities should be made on the report. In this site champion’s context, practice reporting and performance information was considered sensitive due to potentially negative reactions to comparative information by leadership at the site. Therefore the site champion was not interested in testing reports across the professional roles of users to whom the report would be delivered. Furthermore, the site champion suggested that the optimal report would be accompanied by a spreadsheet with the unprocessed count data to support further analyses and follow-up problem solving activities.

A third participant described withholding feedback reports based on their information content to protect team members from burnout and to maintain morale. Given this information in the context of broader sensitivity to performance measurement in the VA, the project team decided to deliver feedback without comparators to increase the likelihood that feedback reports would be delivered to teams at all, and hence to increase their effectiveness.

These differences in responses revealed to the design team that we had falsely assumed that feedback reports would be disseminated to healthcare professionals and teams by site champions at all sites. The suggestion that the reports include additional detailed practice data also increased our understanding of user needs and contextual differences in terms of the purpose of the reports and the nature of problem solving and action planning that might result from the delivery of feedback.

*Example 2: Identifying requirements*

An example of a requirement that held implications for all three refinement steps was the need for reports to distinguish Veterans admitted to short-stay from those receiving long-stay services in Community Living Centers. Early in our iterative testing of reports, participants from some Community Living Centers indicated that they prioritize the conduct of GoCCs differently for Veterans who are not likely to be near end-of-life. For this reason, feedback reports about GoCCs that did not make this distinction in the denominator would be perceived as less appropriate. The implications for refinement of measures was the need to create separate denominators for Veterans admitted to short-stay and long-stay services in Community Living Centers. The implication for data refinement was to determine which clinical services were considered to be short stay and long stay; to identify the data fields in CDW that designated the clinical service at a Veteran’s admission; and to confirm the codes for each admitting service (e.g. hospice, rehab). Then these codes were used to create the new denominators for the measures. The implications for report displays were two-fold: a) in the near-term we decided to exclude short stay Veterans from the displayed charts and revise the report text accordingly b) in the longer-term we decided to design a new chart to accommodate this distinction by constructing two separate charts, one for long stay, the other for short stay.

*Example 3: Refining measures in* Community Living Centers

In the initial design cycle we created a measure of GoCC documentation focusing on timeliness for newly admitted Veterans to Community Living Centers (Table 2). We proposed a numerator to be the number of newly admitted Veterans per quarter who also had a GoCC documented within 7 days following admission to Community Living Centers. Our rationale for focusing on timeliness was that a) the 7-day window represented an early opportunity to capture Veterans’ preferences for care following admission, meaning that the conversation could potentially have the most impact on care provided following admission; b) admission was likely to follow a change in health status that could create a need for reconsideration of care preferences; and c) documentation activity was routinely occurring as part of standard admission procedures, including comprehensive multidisciplinary assessments that are required within the first 7-14 days after admission and d) the 7-day window following admission could serve as a meaningful goal to motivate practice change.

*Design team*

Our design team consisted of a team lead, data analyst, and project assistant (Table). In later stages of the process as the requirements of the design were established, we added a software developer. The design team served a larger implementation project team that was studying the implementation of GoCCs in long term care settings in the Veterans Health Administration[1]. The aim of the design team, through applying the UCD process, was to design a feedback report that could scale to a national level within the Veterans Health Administration.

**Table:** Design team roles and responsibilities

| **Role** | **Key responsibilities** |
| --- | --- |
| Data analyst | - Develop database queries - Identify data quality issues - Refine data |
| Project manager | - Track timelines and tasks - Capture notes during usability testing - Schedule and coordinate usability testing |
| Team lead | - Coordinate assignment of requirements - Conduct usability testing - Refine displays - Refine measures |
| Software developer | - Develop reporting software - Specify report data |

**Reference**

1. Sales AE, Ersek M, Intrator OK, Levy C, Carpenter JG, Hogikyan R, Kales HC, Landis-Lewis Z, Olsan T, Miller SC, Montagnini M, Periyakoil VS, Reder S. Implementing goals of care conversations with veterans in VA long-term care setting: a mixed methods protocol. Implement Sci. 2016 Sep 29;11:132.
