# Supplementary material for: Designing clinical practice feedback reports: three steps illustrated in Veterans Health Affairs long-term care facilities and programs
Source: Implement Sci. 2020 Jan 21;15:7. doi: 10.1186/s13012-019-0950-y (PMC6975062; doi:10.1186/s13012-019-0950-y)
Supplement: Supplementary file 3 — Additional file 3. Software development. Software development process used following the application of the proposed method. [file 13012_2019_950_MOESM3_ESM.docx]

**Additional file 3: Software development**

*Software development*

We transitioned to a software development phase after (a) our observations reached saturation and we were unable to generate any more new insights and (b) usability testing revealed no more significant errors. We developed software tools so that the generation of practice data and practice reports could be done efficiently at large-scale. These tools included a) a program containing database queries to generate practice data (developed in SAS), b) a report generation program to visualize practice data (developed in R) and to generate PDFs with the visualizations and report text (developed in LaTeX). During this stage we continued to seek feedback from participants in phone calls and through follow-up email messages, and to make minor changes to the design of the report based on the feedback we received. After the first version of the software applications for generating reports were complete, we documented the process of report generation into a standard operating procedure and created a quality assessment process for the finished reports, to be conducted prior to their delivery. We developed queries to extract practice data from national VHA databases. During software development, communication about data preparation for reporting increased significantly. We developed a practice data specification to improve the handoff between data extraction and management and report generation tools. We developed the reporting software using a public software repository (GitHub) and made the software available for use using open source software license [https://github.com/Display-Lab/goals-of-care].
